# The effect of metalation on antimicrobial piscidins imbedded in normal and oxidized lipid bilayers†

**DOI:** 10.1039/d3cb00035d

**Published:** 2023-06-07

**Authors:** Ana Dreab, Craig A. Bayse

**Affiliations:** a Department of Chemistry and Biochemistry, Old Dominion University Norfolk VA 23529 USA

## Abstract

Metalation of the N-terminal Amino Terminal Cu(ii)- and Ni(ii)-binding (ATCUN) motif may enhance the antimicrobial properties of piscidins. Molecular dynamics simulations of free and nickelated piscidins 1 and 3 (P1 and P3) were performed in 3 : 1 POPC/POPG and 2.6 : 1 : 0.4 POPC/POPG/aldo-PC bilayers (POPC, 1-palmitoyl-2-oleoyl-*sn*-glycero-3-phosphocholine: POPG, 1-palmitoyl-2-oleoyl-*sn*-glycero-3-phosphoglycerol; aldo-PC, 1-palmitoyl-2-(9′-oxo-nonanoyl)-*sn*-glycero-3-phosphocholine) bilayer models. Nickel(ii) binding decreases the conformation dynamics of the ATCUN motif and lowers the charge of the N-terminus to allow it to embed deeper in the bilayer without significantly changing the overall depth due to interactions of the charged half-helix of the peptide with the headgroups. Phe1⋯Ni^2+^ cation–π and Phe2–Phe1 CH–π interactions contribute to a small fraction of structures within the nickelated P1 simulations and may partially protect a bound metal from metal-centered chemical activity. The substitution of Phe2 for Ile2 in P3 sterically blocks conformations with cation–π interactions offering less protection to the metal. This difference between metalated P1 and P3 may indicate a mechanism by which peptide sequence can influence antimicrobial properties. Any loss of bilayer integrity due to chain reversal of the oxidized phospholipid chains of aldo-PC may be enhanced in the presence of metalated piscidins.

## Abstract 2

Structural changes due to metalation of the ATCUN motif of piscidins embedded in lipid bilayers are revealed through molecular dynamics simulations.

## Graphical abstract

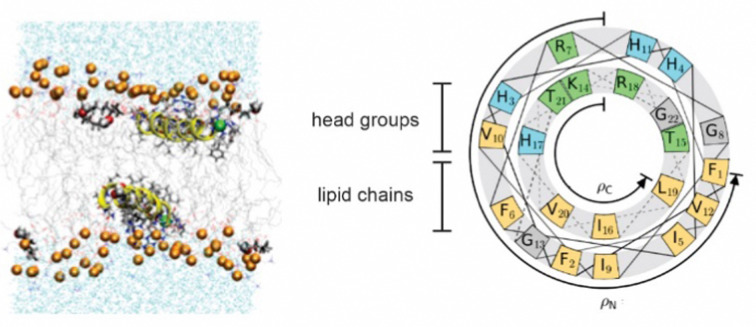

## Introduction

Antibiotic-resistant infections (ARIs) in high-risk patients contribute to significant excess healthcare costs.^1^ Natural or synthetic antimicrobial peptides (AMPs) have been explored to supplement the immune response.^2^ These promising alternatives are considered safe with a low risk of tissue accumulation and bacterial resistance.^3^ AMPs are typically cationic with fewer than fifty amino acids^4^ and classified based on their secondary structure (α-helical, β-sheet, loop, or extended).^5–10^ AMPs strongly bind to bacterial membranes rich in anionic phospholipids but have lower affinity to mammalian cells which have predominantly zwitterionic headgroups. AMP binding is proposed to cause cell leakage and death^2,5^ by altering membrane thickness, promoting phospholipid headgroup clustering, or translocating the membrane to target specific intracellular molecules or processes.^5,6,11–16^ Metal binding is believed to enhance antimicrobial potency.^17–20^ Many AMPs also have complex immunomodulatory functions and synergistic interactions with conventional antibiotics^6^ and several have been approved for clinical use.^2,21^

**Fig. 1:**
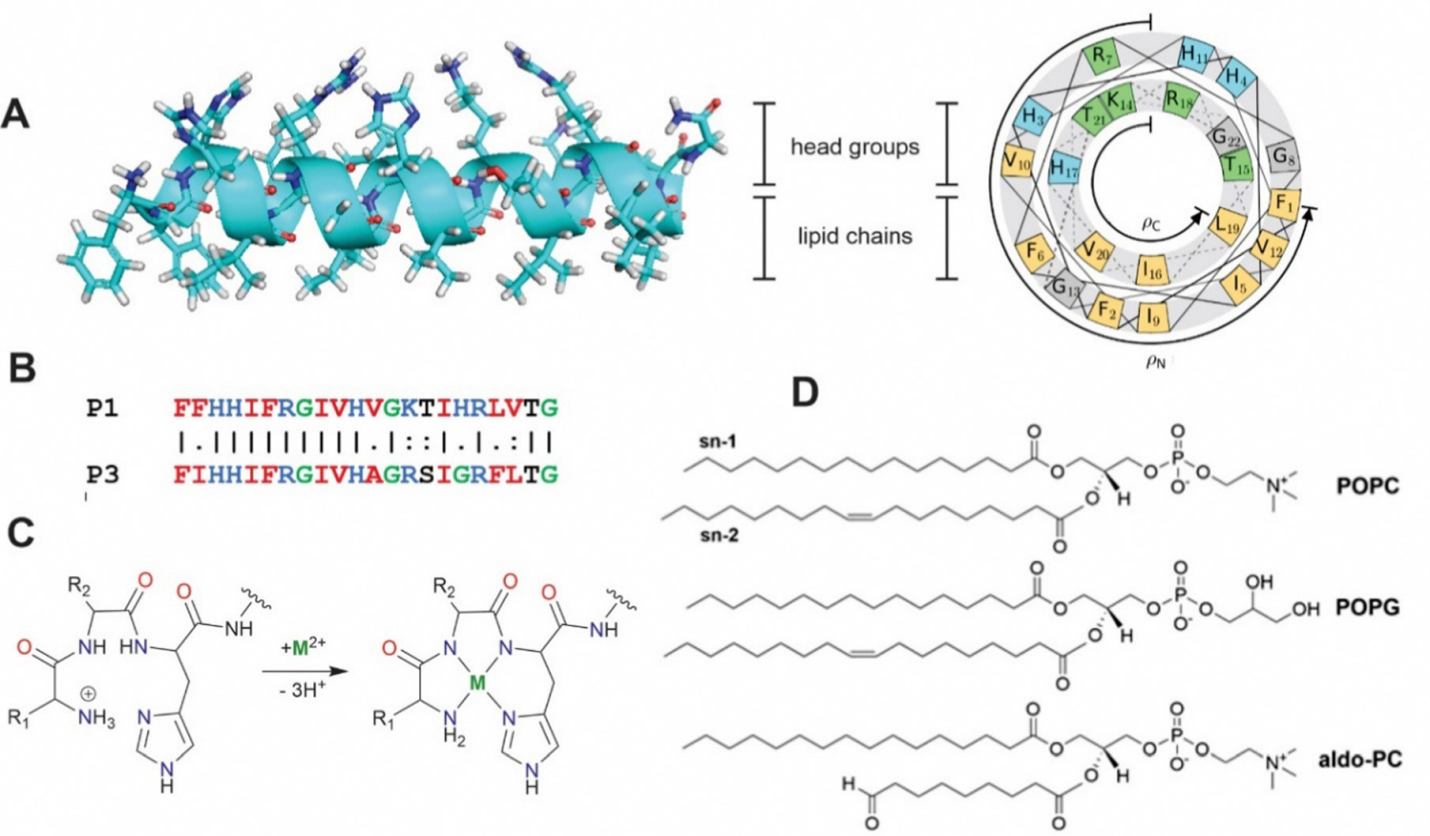
(A) Secondary structure and helical wheel representation (https://helix.perrinresearch.com/wheels/) of P1. The upper polar half helix interacts with the headgroup domain with the non-polar residues of the lower half helix extending into the lipid domain. (B) Sequence alignment of P1 and P3. Basic residues = blue, nonpolar residues = red, Gly = green. (C) Metalation of the ATCUN motif. The net charge of the peptide decreases by one. (D) Phospholipids: POPC = 1-palmitoyl-2-oleoyl-*sn*-glycero-3-phosphocholine, POPG = 1-palmitoyl-2-oleoyl-*sn*-glycero-3-phosphoglycerol, aldo-PC = 1-palmitoyl-2-(9′-oxo-nonanoyl)-*sn*-glycero-3-phosphocholine.

Piscidins, α-helical AMPs found in fish mast cells,^22^ efficiently kill bacteria by permeating and weakening the membrane in a concentration-dependent manner.^23–29^ Piscidins 1 and 3 (P1 and P3, Fig. 1)^22,30^ share 68% sequence identity conserving three histidine residues (His3, His4, and His11).^23,27,31^ An additional His at position 17 of P1 has been proposed to enhance its effectiveness against cancer and HIV.^23,32–35^ Piscidin's N-terminal Amino Terminal Cu(ii)- and Ni(ii)-binding (ATCUN) XXH motif^36^ in particular has a high affinity for binding metals through coordination to the terminal NH_2_, backbone nitrogens of residues 2 and His3, and His3 imidazole-N^δ^. ATCUN-M^2+^ derivatives have been investigated for applications to antitumor activity,^37^ enzyme inhibition,^38^ water oxidation,^39,40^ nitrite reduction,^41^ and imaging agents.^42^ ATCUN-Cu^2+^-containing peptides often have nuclease, protease, and lipase activity which has been harnessed in protein design.^43–45^ The P1 and P3 ATCUN motifs differ at position 2 (Phe *vs.* Ile, Fig. 1B), but each binds Cu^2+^ and Ni^2+^.^17–19,26,43,44^ The enhanced antimicrobial activity of P1/P3:M^2+^ complexes has been attributed to a one-unit reduction in the overall peptide charge through deprotonation of the N-terminal ammonium group (the M^2+^ charge is balanced by the deprotonation of two backbone amides, Fig. 1C).^17,46^ The effect of metalation has been tested using dye leakage assays on synthetic lipid vesicles^31^ with one case resulting in a five-fold increase in permeabilization effectiveness.^47^

Previous molecular dynamics (MD) simulations of the *apo* peptide piscidin embedded in lipid bilayer mimics of bacterial membranes have found bilayer-dependent orientations and insertion modalities.^27,31,48–52^ Additionally, bilayers containing oxidized phospholipids (oxPLs) have been suggested as a novel molecular target for piscidins.^53,54^ The shortened acyl chains of oxPLs terminate in aldehyde or carboxylate groups and can alter the bulk properties of the membrane to increase water content and/or passive permeability.^55–57^ Alternatively, metalated piscidins could generate reactive oxygen species (ROS) locally to oxidize membrane lipids.^38,53,55,58–61^ However, this process may also protect bacteria through changes in membrane structure, biofilm formation, and motility.^59–61^ The behavior of piscidins in these membranes representative of conditions under oxidative stress has not yet been investigated computationally. In this study, MD simulations examine the influence of metalation and oxPLs on the structural behavior of the embedded piscidins P1 and P3 in 3 : 1 POPC/POPG and 2.6 : 1 : 0.4 POPC/POPG/aldo-PC bilayers (POPC = 1-palmitoyl-2-oleoyl-*sn*-glycero-3-phosphocholine, POPG = 1-palmitoyl-2-oleoyl-*sn*-glycero-3-phosphoglycerol; aldo-PC = 1-palmitoyl-2-(9′-oxo-nonanoyl)-*sn*-glycero-3-phosphocholine, Fig. 1D). The ability of P1 to form cation–π and CH–π interactions in the metalated ATCUN motif may contribute to the sequence-dependent performance of these peptides. Chain reversal observed for oxPL may contribute to loss of membrane integrity in the presence of metalated piscidins.

## Computational methods

MD simulations were performed on *apo* and Ni^2+^-bound P1 and P3 in 3 : 1 POPC/POPG and 2.6 : 1 : 0.4 POPC/POPG/aldo-PC lipid bilayers with a ratio of one peptide per 40 lipids in each bilayer leaflet (P/L = 1 : 40) using AMBER18.^62–64^ Model design follows that of Perrin *et al.* using CHARMM.^27^ Simulations of *apo* peptide-containing bilayers provided a benchmark for AMBER simulations with metalated peptides. The 10% aldo-PC mixture has been suggested to be physiologically relevant under conditions of oxidative stress.^65,66^ Simulations of peptide-free bilayers were performed to provide a reference for peptide-induced changes to the membrane. Peptide-bilayer systems were assembled using the Membrane Builder module within the CHARMM-GUI interface.^67,68^ Initial structures were generated as previously described for free piscidins.^27^ The center of mass (COM) of the peptide backbone atoms were aligned ∼20 Å above and below the center of the bilayer. Each peptide was rotated along its helical axis to face the hydrophobic residues toward the bilayer interior. Systems were solvated with 16 Å water layers above each leaflet (∼56 waters/lipid), 15–20 sodium ions, and 0–3 chloride ions to neutralize the models and add a slight salt concentration. Simulations were performed using the *ff14SB*^69^ and Lipid17 force fields.^64^ The aldo-PC force field was derived using the Antechamber module within AMBER18. ATCUN-Ni^2+^ parameters were generated using the Python-based Metal Binding Protein Builder (MCPB) module.^70^ Peptides were modeled with neutral His side chains based upon previously reported p*K*_a_ values.^31^ Solvent and counterions used the TIP3P water model^71^ and Joung–Cheatham monovalent ion parameters.^72^

Models were prepared for production simulations by initial minimization followed by heating from 0 to 313 K using the Langevin thermostat in four stages over 150 ps. Constant volume and weak restraints (force constants of 100 and 10 kcal mol^−1^ Å^−2^ on peptides and lipids, respectively) were applied during the first stage (0 K to 100 K). The remainder of the heating phases (from 100 K to 200 K (2), 200 K to 250 K (3), and 250 K to 313 K (4)) were conducted under constant pressure using semi-isotropic Berendsen regulation with a relaxation time of 2 ps. Production runs were performed at constant pressure (1 atm) and temperature (313 K) in a monoclinic periodic box with zero surface tension using the PMEMD routines.^73–75^ SHAKE constraints were applied to bonds to hydrogen atoms.^76,77^ Electrostatic interactions were treated with the particle mesh Ewald method (cutoff = 10 Å). Since the timescale of peptide-induced defect formation can be from microseconds to minutes,^78^ the MD simulations were performed for 2 μs with a 1 fs time step after 100 ns of equilibration.

Trajectory analysis was performed using Python scripts and the AmberTools CPPTRAJ routines.^79^ Area per lipid (*A*_L_), volume per lipid (*V*_L_), and isothermal compressibility modulus (*K*_A_) were averaged over duplicate 2.0 μs simulations (Tables 1 and 3). *A*_L_, the average area each phospholipid occupies in the *x*- and *y*-plane (eqn (1)), was computed from the dimensions of the periodic box (*L*_*x*_ and *L*_*y*_, respectively) and the number of lipids per leaflet (*m*_lipid_).^80^1
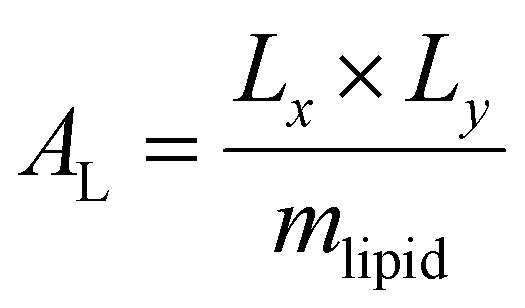
Computational *A*_L_ values were compared to experimental data which can vary depending upon the measurement technique. *V*_L_ was calculated (eqn (2)) from the simulation box volume (*V*_box_), the number of waters (*n*_w_), the volume of a water molecule (*V*_w_ for TIP3P water = 30.53 Å^3^),^81^ and the total number of lipids (*n*_lipid_).^80^2
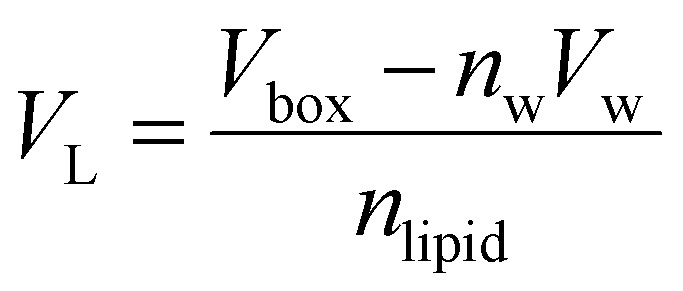
Peptide-induced changes to the fluidity of the bilayer were measured using *K*_A_ (eqn (3)) where *k*_B_ is Boltzmann's constant, *T* is temperature, 〈*A*_L_〉 is the average *A*_L_, and *σ*_A_^2^ is the variance in *A*_L_.^80^3
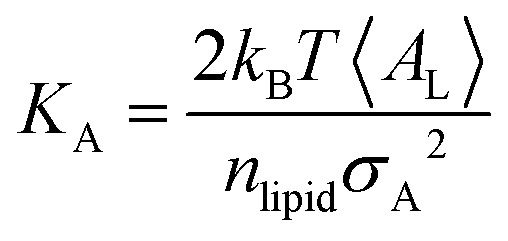
Average electron density profiles for all systems were decomposed into contributions from the following groups: phosphate (PO_4_), methylenes (CH_2_), terminal methyls (CH_3_), and peptide backbone (pepBB) using the density command in CPPTRAJ. The headgroup-to-headgroup (*h*(P–P)) and hydrophobic (*h*(C2–C2)) bilayer thicknesses were determined as the average of the distances between the centers of mass of the phosphate phosphorus and the carbon of the C2 methylene groups from the *sn*-2 chain of each leaflet, respectively. Deuterium order parameters (*S*_CD_, eqn (4))^82^ of the lipid chain carbons reflect the chain mobility at single carbon positions as a measure of the orientation of C–D bonds relative to the *z*-axis. *θ* is the angle between the C–H vector and bilayer normal (*z*-axis) from the simulations, such that an *S*_CD_ value of 1 infers a parallel position and 0 suggests total arbitrary motion.4
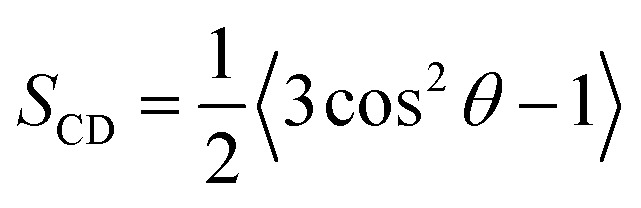
Peptide tilt (*τ*) and azimuthal rotational (*ρ*) angles (Fig. 1A) were determined at 10 ns intervals relative to a reference structure consisting of α-helix *φ*/*ψ* angles of −61°/−45° and Euler angles of zero. Peptide insertion depths (*z*) were calculated as the distance between the COM of the peptide backbone heavy atoms and the plane of the phosphorus atoms of the lipid headgroups. *τ*, *ρ* and *z* were calculated separately for the N-terminal (residues 5–10) and C-terminal (residues 14–20) ends due to fraying of terminal residues, metalation of the N-terminus, and previously reported kinking at a flexible helix turn that includes G13.^27^

**Table:** Average structural properties of the POPC/POPG bilayer model (*V*_L_ = volume per lipid, *A*_L_ = area per lipid, *K*_A_ = isothermal area expansion modulus, *h*(P–P) = headgroup-to-headgroup thickness, *h*(C2–C2) = hydrophobic thickness)

POPC/POPG	*V*_L_ (Å^3^)	*A*_L_ (Å^2^)	*K*_A_ (mN m^−1^)	*h*(P–P) (Å)	*h*(C2–C2) (Å)
Exp	*1256* b	*64.3^b^, 65.3* c, *68.3*^b^	*180*–*330*^a^	*37.0* b	
	*1265* c	67.1 ± 0.4		3*6.0*^c^	
	1206 ± 0			37.5 ± 0.2	27.4 ± 0.2
MD			254 ± 114	*39.0 ± 1.0*	*28.9 ± 0.7*
+P1	1290 ± 0	72.1 ± 0.5	208 ± 87	37.3 ± 0.4	27.4 ± 0.4
			*332 ± 42*	*37.4 ± 1.2*	*28.0 ± 1.2*
+P3	1287 ± 1	71.8 ± 0.6	218 ± 120	37.2 ± 0.3	27.3 ± 0.4
			*248 ± 22*	*37.0 ± 1.1*	*27.5 ± 1.1*
+P1:Ni	1290 ± 0	71.9 ± 0.4	202 ± 98	37.4 ± 0.3	27.5 ± 0.3
+P3:Ni	1286 ± 0	71.4 ± 0.4	250 ± 98	37.5 ± 0.3	27.6 ± 0.3

a The experimental value for pure POPC at 298 K.^83^

b The experimental value for pure POPC at 303 K.^84^

c The experimental value for pure POPC at 313 K.^85^

## Results and discussion

### MD simulations of apo piscidins in POPC/POPG bilayers

**Fig. 2:**
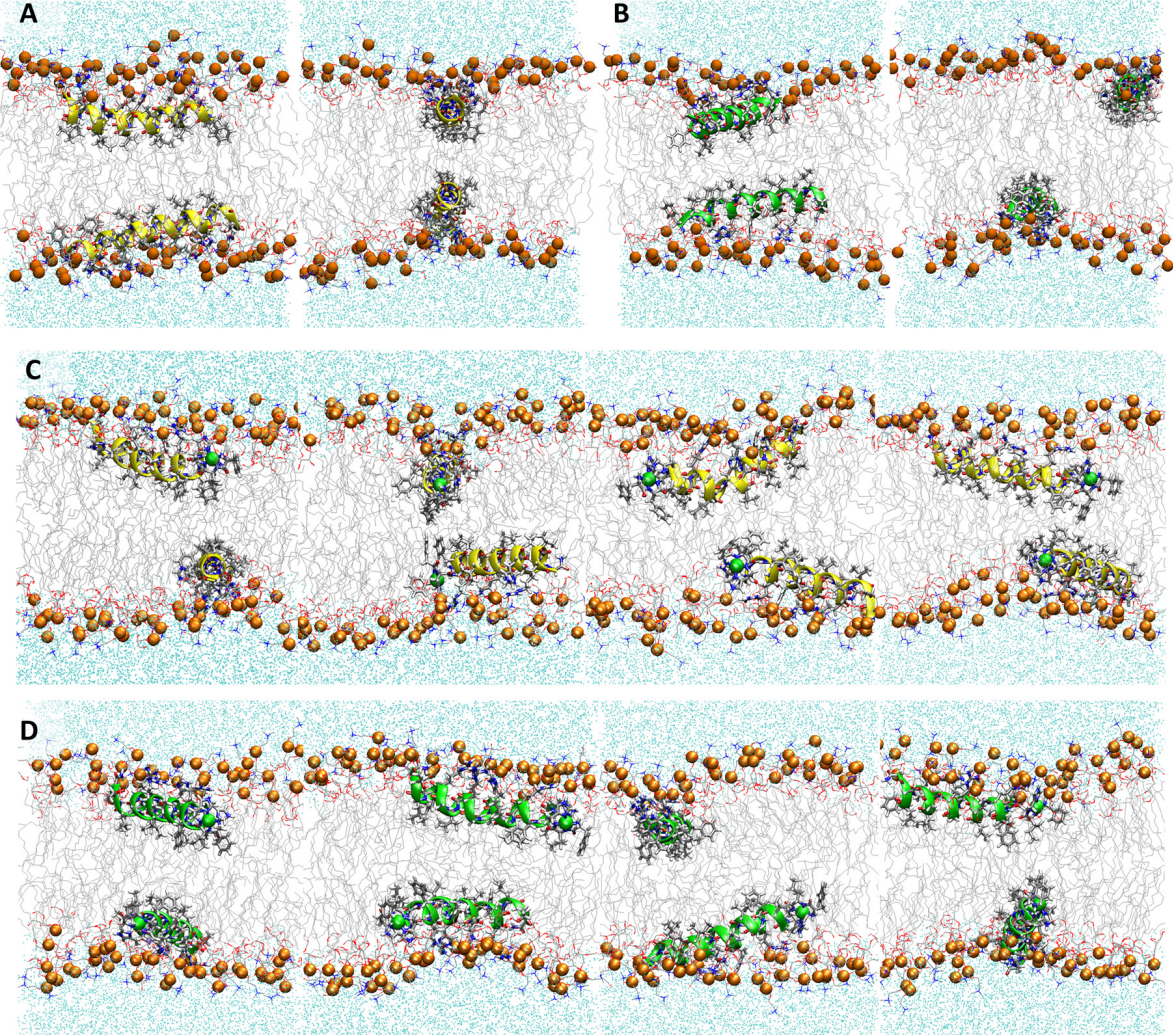
Snapshots from MD simulations of (A) P1, (B) P3, (C) P1:Ni, and (D) P3:Ni in the POPC/POPG bilayer model. Peptides are generally localized at the headgroup/lipid sublayer interface and do not interact with one another. Peptides are represented as yellow (P1 isoforms) or green (P3 isoforms) ribbons and licorice. Phosphorus atoms are depicted as orange spheres while the rest of phospholipids atoms except hydrogen are depicted as lines colored as follows: carbon – gray, oxygen – red, and nitrogen – blue. Water molecules are represented as cyan points.

**Fig. 3:**
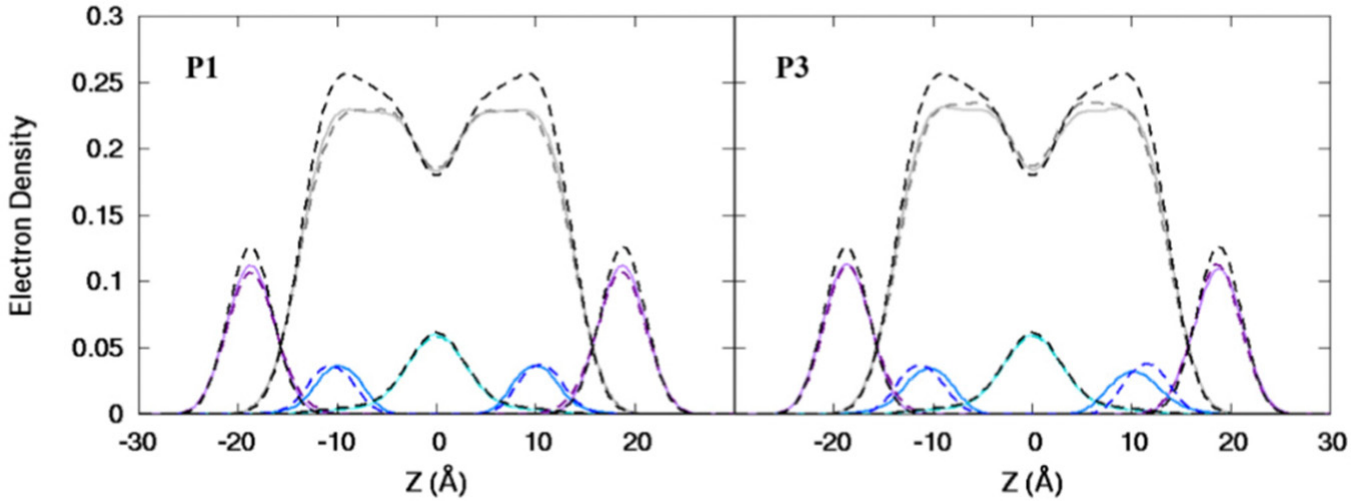
Electron density profiles for P1 and P3 in the 3 : 1 POPC/POPG bilayer. Profiles are shown for the phosphates (purple), peptide backbones (blue), CH_2_ groups (gray), and terminal CH_3_ (cyan). Electron density profiles for *apo* or metalated piscidin-bound bilayer are in dashed or solid lines, respectively, whereas the bilayer-only systems are in dashed black lines.

MD simulations of peptide-free, P1-, and P3-bound 3 : 1 POPC/POPG bilayers performed with AMBER are generally consistent with previous models in CHARMM.^48^ Embedded P1 and P3 maintain their α-helical structure throughout simulations with the average orientation of their N-termini canted slightly toward the headgroups (*τ*_N_ ≈ 95°) and the C-terminal sequences nearly parallel to the surface (*τ*_C_ ≈ 89°/88°, Fig. 2 and S1 (ESI†), Table 2).^27^ The peptide backbones for P1 and P3 float on average ∼7–8 Å below the P atoms, just under the C2 atoms of the lipid *sn*-2 acyl chain (Fig. 3 and Table 2). P1 inserts slightly deeper in the bilayer *versus* P3 but both experience similar differences in the *z*_N_ and *z*_C_ insertion depth values (Δ*z* ∼ 0.1 Å). P3 is slightly more flexible than P1 based on the backbone heavy-atom RMSD of the first 20 residues (0.98 Å *vs.* 0.80 Å, respectively).

Average tilt angles-*τ*, azimuthal rotation angles *ρ* (Fig. 1A), and depths of insertion *z* for the backbone atoms of N-(residues 5–10), C-(residues 14–20) helical segments, and the full peptide in POPC/POPG bilayer models

**Table:** 

	*τ*_N_, °	*τ*_C_, °	Δ*τ* = (*τ*_N_ − *τ*_C_), °
P1	95 ± 1	88 ± 1	7.5 ± 1
P3	95 ± 1	89 ± 1	6.1 ± 0
P1:Ni	90 ± 1	83 ± 1	7.0 ± 1
P3:Ni	86 ± 2	82 ± 2	4.0 ± 1

**Table:** 

	*ρ*_N_, °	*ρ*_C_, °	Δ*ρ* = (*ρ*_N_ – *ρ*_C_), °
P1	261 ± 2	241 ± 1	19.5 ± 1
P3	248 ± 1	233 ± 2	15.3 ± 0
P1:Ni	251 ± 1	235 ± 1	16.0 ± 1
P3:Ni	237 ± 1	224 ± 1	12.7 ± 1

**Table:** 

	*z*_N_, Å	*z*_C_, Å	Δ*z* = (*z*_N_ − *z*_C_), Å	*z*_pep_, Å
P1	−8.2 ± 0.2	−8.3 ± 0.3	0.1 ± 0.2	−7.8 ± 0.3
P3	−7.4 ± 0.1	−7.5 ± 0.3	0.1 ± 0.2	−7.0 ± 0.2
P1:Ni	−9.1 ± 0.2	−8.0 ± 0.3	−1.2 ± 0.1	−8.2 ± 0.2
P3:Ni	−9.1 ± 0.4	−7.1 ± 0.2	−2.0 ± 0.5	−7.9 ± 0.2

Attractive interactions with the PO_4_ groups may contribute to the asymmetric widening of their density peaks found in both the current and previous simulations (Fig. 3).^48^*A*_L_, *V*_L_, and *K*_A_ values indicate that the free bilayer is equilibrated in the liquid phase and consistent with experimental measurements of a single component POPC bilayer (Table 1).^83–85^ Interactions of the peptides with the phospholipid headgroups and upper chain regions induce increases in *A*_L_ and *V*_L_ as well as greater bilayer fluidity as measured by *K*_A_, which contrasts with previous results that found that *K*_A_ decreased in the presence of P3 but increased upon addition of P1.^48^ Looser packing of aliphatic lipid chains (lower *K*_A_) was also observed in the MD simulations of fentanyl in a 1,2-dioleoyl-*sn*-glycero-phosphocholine (DOPC) bilayer.^81^ P1 and P3 display similar *ρ*_C_ angles with a smaller *ρ*_N_ for the latter (248° *vs.* 261°, Table 2), indicating that the P3 N-terminal sequence distributes its hydrophobic and hydrophilic residues more unevenly in the bilayer. Similar results have been reported for P1 and P3 in DMPC/DMPG and POPE/POPG bilayers (Δ*τ* ∼ 6–8° and Δ*ρ* ∼ 15–20°).^27^ The differential rotation of the two piscidin segments maximizes hydrophobic interactions with the nonpolar half helix settled into the lipid sublayer. Residues of the polar half helix are solvent-exposed through a gap in the headgroup sublayer with the sidechains of Lys and Arg extended to salt bridge with anionic phosphates. Water molecules penetrate into the headgroup sublayer but are rarely found below the peptide within the nonpolar region of the bilayer. Examples of “funneling” as observed in previous studies^31^ were short-lived and not accompanied by movement of water through the bilayer, although frames with single water molecules in the nonpolar domain could be occasionally observed.

The *apo* piscidins induce a slight thinning of the bilayer (< 0.3 Å) as calculated by the *h*(P–P) distances with little effect on the *h*(C2–C2) thickness (Table 1). In contrast, the *h*(C2–C2) values for P1 and P3 decrease by 0.9 Å and 1.4 Å, respectively, in CHARMM simulations^48^ where the standard errors (±1.1–1.2 Å) suggest that piscidins cause large variations in the hydrophobic thickness of the membrane. However, the free bilayer in our AMBER simulations is on average ∼1.5 Å thinner than those modeled with the CHARMM force fields (*i.e.*, *h*(P–P) ∼37.5 Å and ∼39.0 Å, respectively, Table 1).^48^ This difference in results could be attributed to short simulation times in CHARMM (100 ns) and/or a force field dependence on the results of membrane-bound AMP simulations.^86^ In either case, the 1 : 40 model with the placement of peptides on both sides of the leaflet may be too small and symmetric to observe bulk distortions that could be attributed to membrane disruption. Therefore, in the following discussion, we instead focus on the local effects of metalation on the peptide and bilayer.

### MD simulations of metalated piscidins in POPC/POPG bilayers

**Fig. 4:**
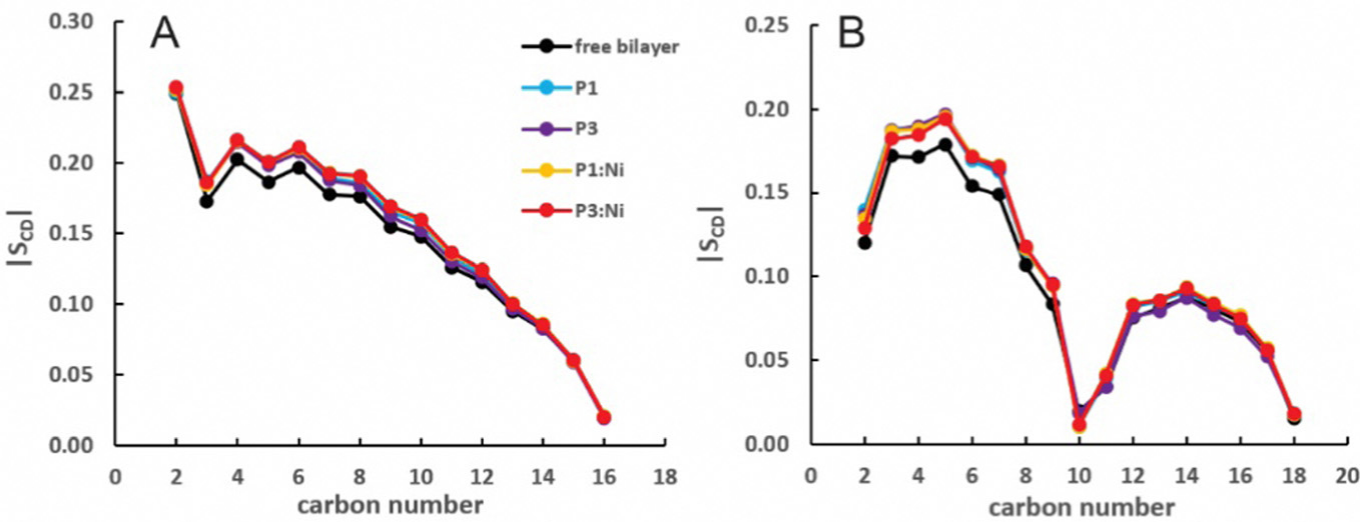
Average |*S*_CD_| for the (A) *sn*-1 and (B) *sn*-2 lipid acyl chains for the free 3 : 1 POPC/POPG bilayer and in the presence of P1 (cyan), P3 (purple), P1 : Ni (orange), and P3 : Ni (red).

Nickelated P1 and P3 embed into the 3 : 1 POPC/POPG bilayer similarly to the *apo* peptides maintaining their highly helical structure (RMSD = 1.07 Å and 1.13 Å, respectively) with similar *h*(P–P) and *h*(C2–C2) thickness to the *apo* peptide simulations (Table 1). The metalated ATCUN motif is less dynamic (RMSD = 0.19 Å and 0.22 Å, respectively, Table 2) than the *apo* piscidin N-termini due to the rigid Ni^2+^ coordination sphere. A decrease in *K*_A_ for P1:Ni suggests an increase in membrane fluidity, where P3:Ni shows little change compared to the bilayer alone. Charge neutralization of the ATCUN-Ni^2+^ motif contributes to deeper embedding of the N-termini relative to the *apo* peptide by ∼0.9 Å for P1:Ni and ∼1.7 Å for P3:Ni (|*z*_N_ − *z*_C_|, 1–2 Å (metalated) *vs.* 0.1 Å (*apo*), Table 2 and Fig. S1, ESI†). The mean peptide distance from the bilayer center increases as P1:Ni < P3:Ni < P1 < P3 with P1:Ni displaying a density maximum at ∼9.9 Å. Similar bilayer behavior is also observed upon cholesterol incorporation into the membrane.^87,88^ The N-terminus is more parallel to the bilayer surface for P1:Ni and slightly canted toward the bilayer center (*τ*_N_ = 86°) in P3:Ni with the C-termini tilted toward the bilayer core (Table 2). Nickelated P1/P3 adopt *ρ*_N_ smaller values by 10° and 11°, respectively, relative to the *apo* peptides, with Δ*ρ* (*ρ*_N_ − *ρ*_C_) lower by only ∼3.5° upon metal-binding for the following trend in Δ*ρ*: P1 > P1:Ni ≈ P3 > P3:Ni (Table 2). These distribution angles allow better positioning of Phe1 and Phe2/Ile2 for interaction with the lipid hydrophobic core and of His3 with the water-bilayer interface. However, attractions between the hydrophilic residues of the polar half helix and the solvent and headgroups prevent a lower depth of insertion. This enhanced N-terminal insertion may contribute to the higher toxicity of metalated piscidins toward planktonic bacteria.^26^ The *S*_CD_ order parameter of the piscidin-embedded bilayers (Fig. 4) indicate increased order in the acyl *sn*-1 and *sn*-2 chains nearest the headgroups. This restricted motility induced by the peptides decreases along the chains but does not discriminate between isoforms (P1 or P3) or metalation state (*apo* or Ni^2+^-bound).

### MD simulations of free POPC/POPG/aldo-PC bilayers

**Fig. 5:**
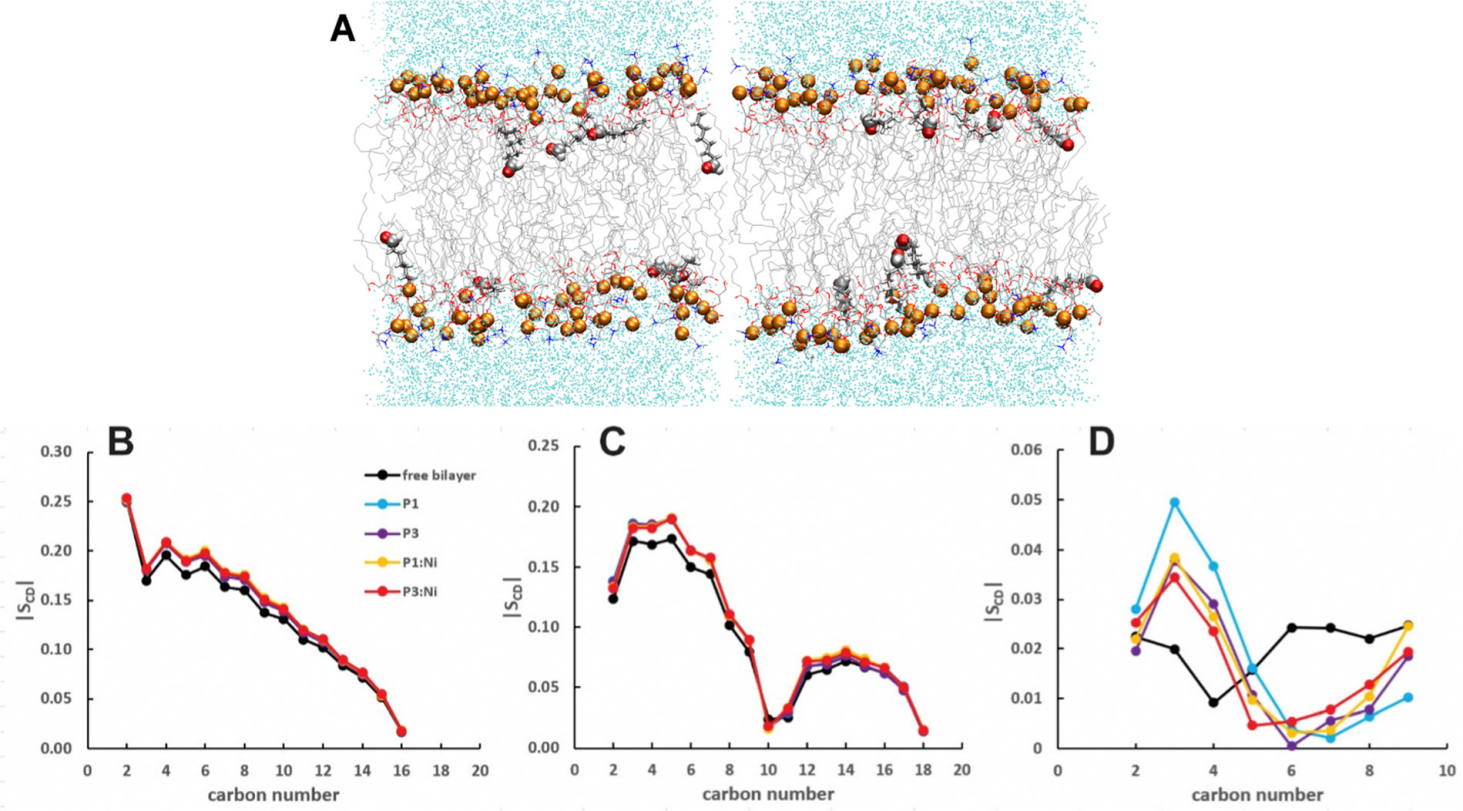
Snapshots from MD simulations of POPC/POPG/aldo-PC illustrating that oxPL chains can organize parallel to the headgroup region. Aldo *sn*-2 acyl chains are represented as licorice and the carbonyl group as spheres. Average |*S*_CD_| for the (B) *sn*-1, (C) *sn*-2 lipid, and (D) oxPL acyl chains for the free POPC/POPG/aldo-PC bilayer and in the presence of P1 (cyan), P3 (purple), P1:Ni (orange), and P3:Ni (red).

The 2.6 : 1 : 0.4 POPC/POPG/aldo-PC bilayers are thinner by ∼1 Å (both *h*(P–P) and *h*(C2–C2)) for a smaller *V*_L_ (1183 Å^3^) (compare Tables 1 and 3) relative to POPC/POPG due to the shortened oxPL chains. *A*_L_ is similar to the 3 : 1 POPC/POPG bilayer with a lower *K*_A_ (243 ± 88 *vs.* 254 ± 114), consistent with greater fluidity due to the 4 short aldo-PC chains per leaflet. A more fluid membrane (lower *K*_A_ and *S*_CD_) containing kinked lipid tails (smaller *S*_CD_) is consistent with the potential for oxPLs to disrupt membrane bulk proprieties for enhanced permeabilization.^55–57^ The oxPL chains can occasionally be found oriented parallel to the headgroup region to allow the aldehyde tails to interact with the more polar headgroups (Fig. 5A). While these chain reversals may contribute to membrane instability, they did not disrupt bilayers in simulations of up to 25% oxPL.^54^

**Table:** Average structural properties of the POPC/POPG/aldo-PC bilayer model (*V*_L_ = volume per lipid, *A*_L_ = area per lipid, *K*_A_ = isothermal area expansion modulus, *h*(P–P) = headgroup-to-headgroup thickness, *h*(C2–C2) = hydrophobic thickness)

	*V*_L_ (Å^3^)	*A*_L_ (Å^2^)	*K*_A_ (mN m^−1^)	*h*(P–P) (Å)	*h*(C2–C2) (Å)
Exp	*1256* a			*37.0* a	
	*1265* b	*64.3^a^, 65.3* b *68.3* a	*180*–*330*^a^	3*6.0*^b^	
MD	1183 ± 0	67.7 ± 0.6	243 ± 88	36.4 ± 0.2	26.3 ± 0.3
+P1	1267 ± 0	72.5 ± 0.5	185 ± 81	36.3 ± 0.3	26.3 ± 0.5
+P3	1264 ± 0	72.1 ± 0.5	198 ± 95	36.3 ± 0.4	26.4 ± 0.5
+P1:Ni	1267 ± 0	72.2 ± 0.4	222 ± 93	36.5 ± 0.3	26.6 ± 0.3
+ P3:Ni	1264 ± 0	71.9 ± 0.5	212 ± 86	36.5 ± 0.3	26.5 ± 0.4

a The experimental value for pure POPC at 303 K.^84^

b The experimental value for pure POPC at 313 K.^85^

### MD simulations of *apo*- and metalated piscidins in POPC/POPG/aldo-PC bilayers

**Fig. 6:**
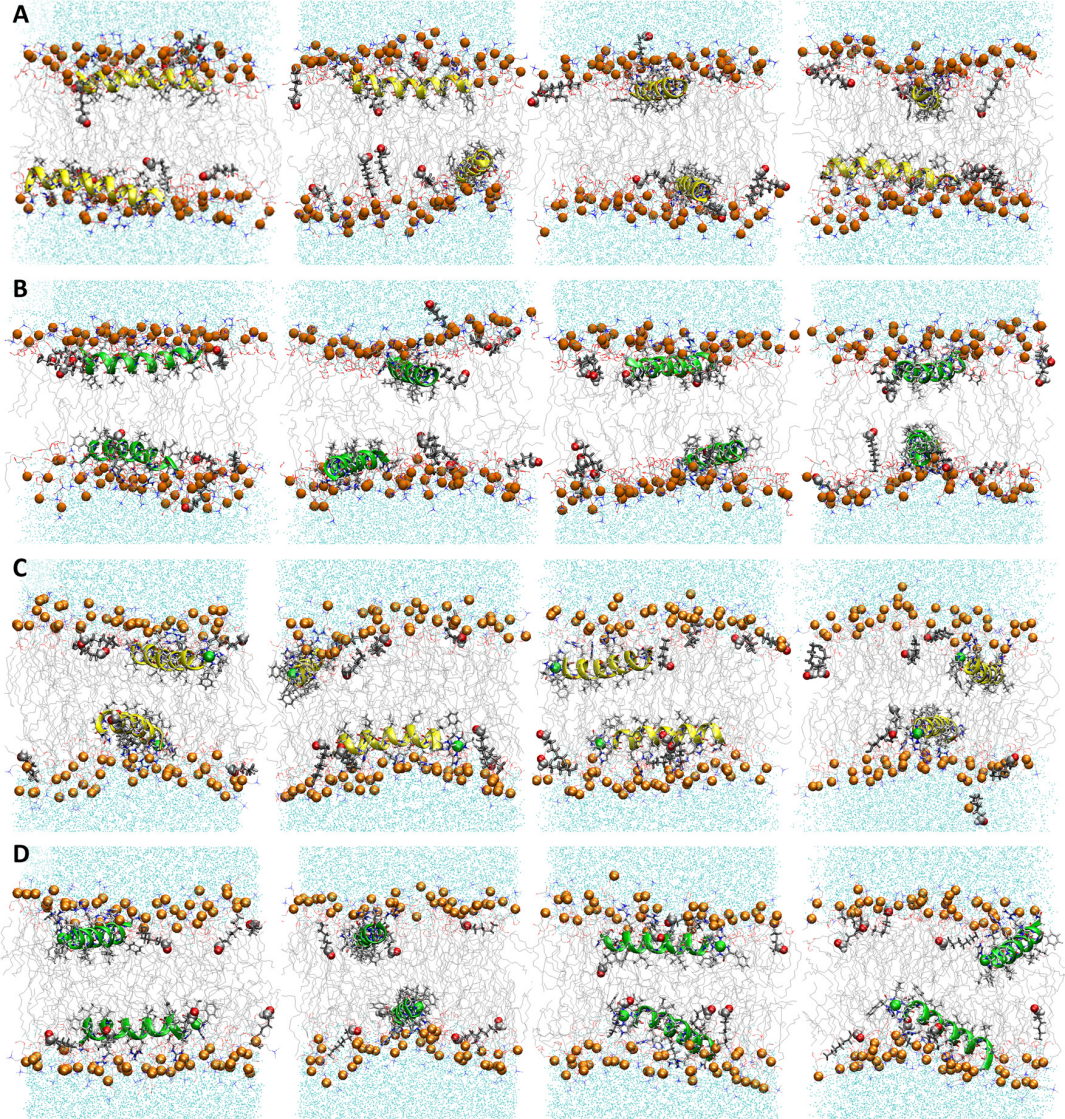
Snapshots from MD simulations of (A) P1, (B) P3, (C) P1:Ni and (D) P3:Ni in POPC/POPG/aldo-PC. Peptides are generally localized at the headgroup/lipid sublayer interface and do not interact with one another. Peptides are represented as yellow (P1 isoforms) or green (P3 isoforms) ribbons and licorice. Ni^2+^ ions and phosphorous atoms are depicted as green and orange spheres, respectively, while the rest of the phospholipid atoms except for hydrogen are depicted as lines colored as follows: carbon – gray, oxygen – red, and nitrogen – blue. Aldo *sn*-2 acyl chains are represented as licorice, and water molecules as cyan points.

The α-helices of the *apo* and metalated piscidins settle below the phosphate plane of the aldo-PC-containing bilayer (RMSD = 0.75/0.78 Å (P1/P1:Ni) and 0.83/0.89 Å (P3/P3:Ni)) with low RMSD values for the ATCUN residues (0.20 Å (P1:Ni) and 0.22 Å (P3:Ni)) (Fig. 6). The *apo* piscidins orient their N-termini toward the headgroups (*τ* = 95°/93) with *ρ* values of 259° (P1) and 249° (P3) (Table 4). The increased *A*_L_ for the *apo* piscidin-embedded bilayer, with a slightly more pronounced expansion for the P1 isoform (Table 3), could be associated with a more dynamic diffusion of the peptide molecules through the bilayer. *V*_L_ is slightly smaller relative to the POPC/POPG bilayers due to the shortened oxPL chain (Tables 1 and 3). The embedded peptides disrupt phospholipid packing for a ∼9–24% drop in fluidity as measured by *K*_A_ (Table 3) with *apo* peptides lower than the metalated piscidins and P1:Ni having the lowest decrease in *K*_A_ relative to the POPC/POPG bilayer. While the average number of hydrogen bonds between the PC and PG headgroups is generally lower in the presence of piscidin, P1:Ni (and P3:Ni in POPC/POPG) induced only a slight decrease in hydrogen bonding (Fig. S3, ESI†).

Average tilt angles *τ*, azimuthal rotation angles *ρ* (Fig. 1A), and depths of insertion *z* for the backbone atoms of N-(residues 5–10), C-(residues 14–20) helical segments, and the full peptide in POPC/POPG/aldo-PC bilayer models

**Table:** 

	*τ*_N_, °	*τ*_C_, °	Δ*τ* = (*τ*_N_ – *τ*_C_), °
P1	95 ± 1	88 ± 1	7.6 ± 1
P3	93 ± 0	89 ± 1	4.5 ± 1
P1:Ni	90 ± 1	83 ± 1	6.6 ± 1
P3:Ni	85 ± 1	83 ± 1	2.1 ± 1

**Table:** 

	*ρ*_N_, °	*ρ*_C_, °	Δ*ρ* = (*ρ*_N_ − *ρ*_C_), °
P1	259 ± 2	240 ± 2	19.0 ± 1
P3	249 ± 3	228 ± 4	21.0 ± 2
P1:Ni	251 ± 1	234 ± 2	16.3 ± 1
P3:Ni	232 ± 4	216 ± 2	16.0 ± 6

**Table:** 

	*z*_N_, Å	*z*_C_, Å	Δ*z* = (*z*_N_ − *z*_C_), Å	*z*_pep_, Å
P1	−8.1 ± 0.2	−8.2 ± 0.4	0.2 ± 0.2	−7.7 ± 0.2
P3	−7.6 ± 0.1	−7.6 ± 0.1	0.0 ± 0.0	−7.2 ± 0.1
P1:Ni	−9.2 ± 0.3	−8.0 ± 0.3	−1.2 ± 0.1	−8.4 ± 0.3
P3:Ni	−8.7 ± 0.2	−6.9 ± 0.2	−1.8 ± 0.2	−7.6 ± 0.1

The N-terminus of P1:Ni generally orients parallel to the headgroups with P3:Ni slightly canted toward the bilayer core (*τ*_N_ = 85°) for (Table 4 and Fig. S2, ESI†). The C-termini tilt toward the bilayer center for the Ni^2+^-bound piscidins or parallel to the surface for the *apo* piscidins. The P1 rotation angles *ρ* are affected by metal binding as P1:Ni has similar values in both lipid mixtures. The P3:Ni *ρ* values are more responsive to metalation and oxPL as *ρ*_N_ and *ρ*_C_ decrease by 5° and 8°, respectively, and sample a broader range (Tables 2 and 4, and Fig. S2, ESI†) for a more uneven distribution of the C-terminal hydrophobic and hydrophilic residues in the bilayer. P3 is the only isoform where Δ*ρ* is appreciably different in the presence of aldo-PC (+5.7° and +3.3° for the *apo* and Ni^2+^-bound piscidin, respectively) with the following Δ*ρ* trend for the studied peptides: P3 > P1 > P1:Ni ≈ P3:Ni.

**Fig. 7:**
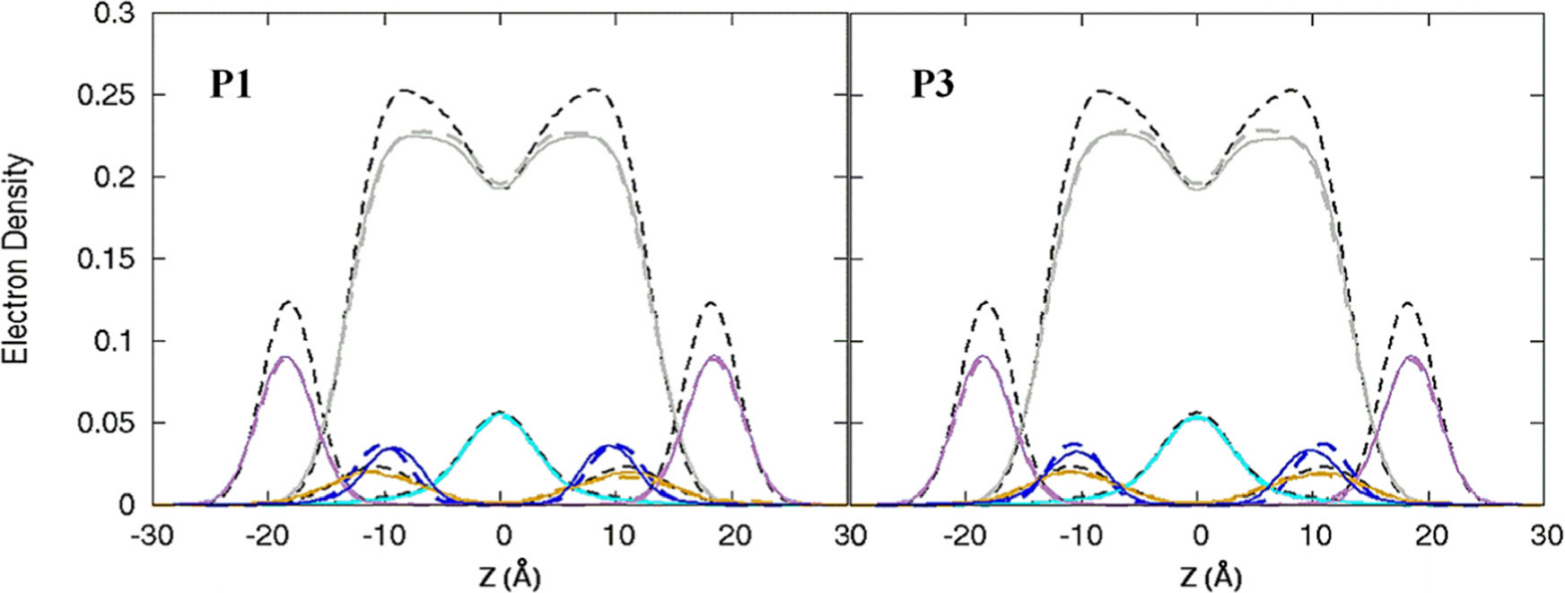
Electron density profiles for P1 and P3 in POPC/POPG/aldo-PC. Profiles are shown for the phosphates (purple), peptides (blue), CH_2_ acyl chains (gray), and terminal CH_3_ (cyan). Electron density profiles containing membrane and *apo* or metalated piscidin are in dashed or solid lines, respectively, whereas the systems with only the membrane are in dashed black lines.

The peptides are located at similar depths to the POPC/POPG bilayer (Table 4) and sample a relatively narrow *z*_N_ range. P1 inserts embeds ∼0.5 Å deeper into the oxPL membrane than P3, similar to the behavior in POPC/POPG bilayer reported in this study and previously by Perrin *et al.*^48^ (Table 4). Metalation increases the insertion of the N-termini by ∼1.1 Å such that *z*_N_ of P1:Ni settles deeper in the bilayer than P3:Ni (*z*_N_ ∼ 9.2 Å *vs.* 8.7 Å). Meanwhile, the peptides’ C-termini, particularly of P3:Ni, insert less with smaller |Δ*z*| than their *apo* counterparts. The incorporation of oxPL into the bilayers does not significantly affect the electron density profiles relative to the POPC/POPG systems (Fig. 7) except for a larger decrease in the PO_4_ density peak in the POPC/POPG/aldo-PC bilayers. The maximum density of P1:Ni places it closest to the bilayer center (∼9.3 Å *vs.* ∼9.9 Å in POPC/POPG). The *h*(P–P) and *h*(C2–C2) bilayer thicknesses are similar to those observed for POPC/POPG membranes.^48^ Where the oxPL-containing bilayer is slightly thinner with embedded *apo* piscidins based on the *h*(P–P) distance, metalation leads to a slight thickening in C2–C2 because deeper insertion reverses the contraction of the bilayer due to the lower volume of oxPL (Table 3). The trends in the |*S*_CD_| of the *sn*-1 and *sn*-2 chains (Fig. 5) are similar to the POPC/POPG bilayer results and not significantly influenced by the presence of the aldo-functionalized *sn*-2 tails. These shortened chains occasionally snorkel toward the water layer to interact with the positively charged trimethylamino groups of the headgroup domain when piscidins are embedded (*i.e.*, Fig. 5B–D) as indicated by the change in pattern of |*S*_CD_| relative to the free bilayer.

The PG headgroup region responds more strongly to P1 : Ni in the presence of aldo-PC and at the 1 : 20 peptide-to-lipid ratio in ^31^P NMR studies.^47^ In simulations, the interactions between piscidin and PC/PG headgroups assessed through hydrogen bond analysis do not necessarily reflect a peptide preference for a specific headgroup. Nonetheless, the average number of PG-peptide hydrogen bonding interactions increases slightly for P1:Ni, but not for P3:Ni, in the presence of aldo-PC, in agreement with the experimental data (Fig. S4, ESI†).^47^*Apo* P1 increases its hydrogen bonding with the PC headgroups, and reduces those with PG, whereas P3 follows an opposite trend. Altogether, P1:Ni interacts more readily with the anionic PG headgroups, which correlates well with the enhanced membrane activity of the peptide on the POPC/POPG/aldo-PC lipid mixture.^47^

### Conformation dynamics of the ATCUN:Ni^2+^ motif

**Fig. 8:**
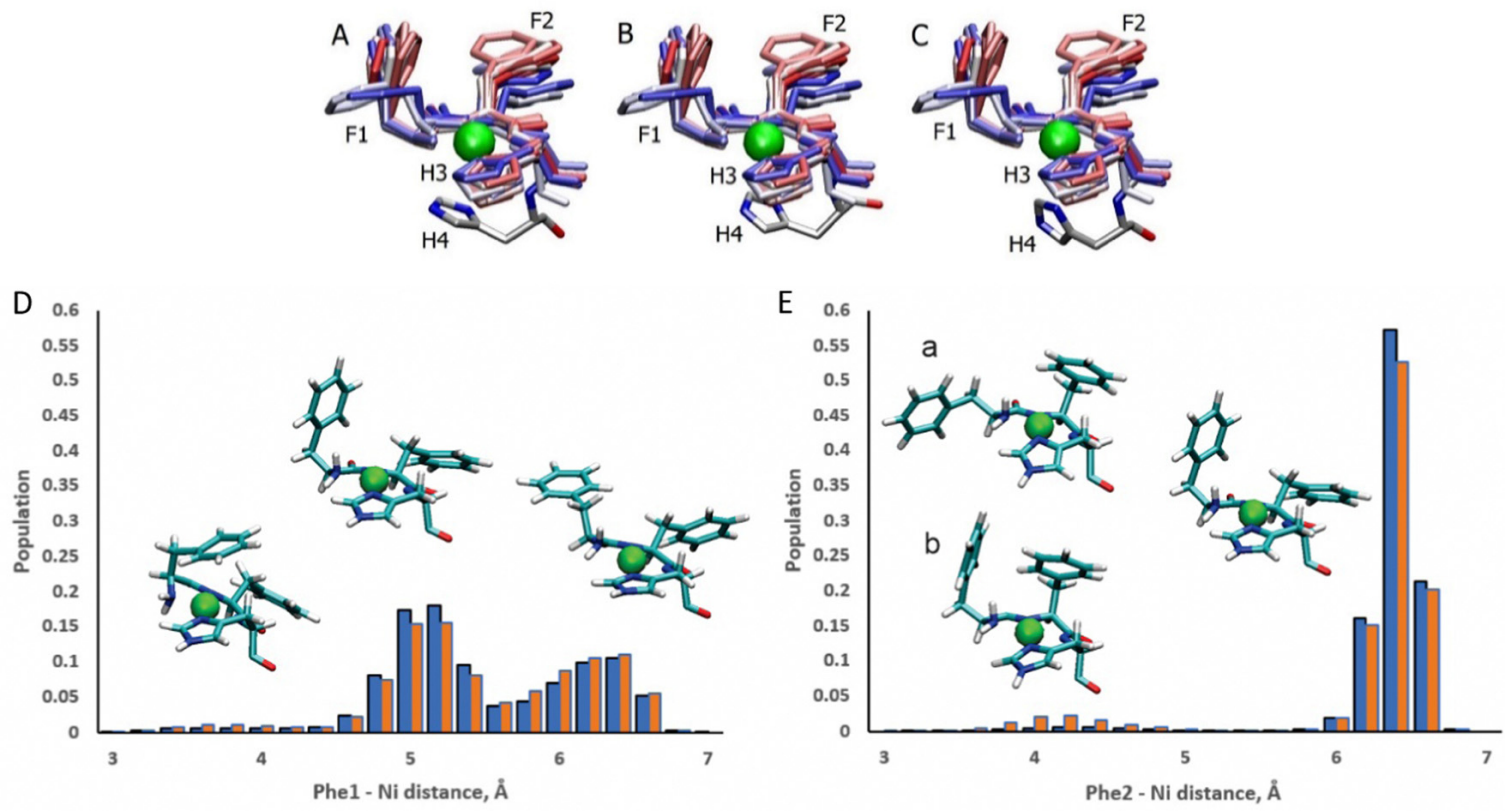
Snapshots of the P1:Ni ATCUN residues 1–3 in POPC/POPG taken from the MD simulations at intervals of 200 ns (0 : 20 : 209). Color-coded with red at the beginning of the trajectory and blue at the end. H4 residue is included at *t* = 1100 ns (A), 1700 ns (B), and 2100 ns (C). Population distributions with representative structures of the ATCUN motif for the distances between the Ni^2+^ ion and (D) the COM of the Phe1 phenyl ring and (E) the COM of the Phe2 phenyl ring of P1 in the POPC/POPG (blue) and POPC/POPG/aldo-PC (red) bilayer models. Structures labeled (a) and (b) are representative of snapshots with short Phe2–Ni^2+^ distances. Structure (a) is consistent with a Phe2–Ni^2=^ cation–π interaction. In structure (b) Phe2 has a short contact with Ni^2+^, but a cation–π interaction with Phe1.

The ATCUN motif binds metals through the N-terminal amine, the deprotonated backbone amides of residues 2 and His3, and the His3 imidazole group. Metal binding allows the piscidins to embed deeper in the bilayer and decreases the conformational dynamics of the N-terminus. The Ni^2+^ ion adopts square-planar coordination which leaves two potential open coordination sites. In simulations, a fifth site is occupied by the His4 imidazole facing the headgroup region in 94% of frames leaving the sixth site embedded in and generally exposed to the nonpolar sublayer. The aromatic sidechains of the P1 and P3 ATCUN motif have been proposed to stabilize the Ni^2+^ ion through cation–π interactions at the sixth coordination site to aid insertion of the metal-bound N-termini into the bilayer.^89^ The COM of the P1 Phe2 phenyl ring is positioned ∼6.5 Å from the metal for 96% of frames while Phe1 makes closer contacts of ∼5.2 Å for 59% (Fig. 8). Although these distances are within the range expected for cation–π interactions,^90,91^ their actual orientations are generally not consistent with formation of a strong attractive interaction with the metal. A fully extended conformation with long COM-Ni^2+^ distances for both residues (∼6.3 for 37% of frames) occurs when the Phe1 sidechain protrudes into the lipid bilayer and Phe2 folds back to interact with Phe6. However, Phe1 and Phe2 are found to intermittently make a much closer contact of ∼4.0 Å and ∼4.2 Å, respectively, for ∼3% and ∼2.5% of frames in POPC/POPG, respectively, consistent with partial shielding of the metal by aromatic sidechains.^89^ The populations of these close contacts increase to ∼4% (Phe1) and ∼8% (Phe2) in POPC/POPG/aldo-PC due to the greater fluidity of the oxPL-containing bilayer. Short Phe2-Ni^2+^ distances can be divided into a potential cation–π-type interactions (a in Fig. 8E) and close contacts due to a CH–π interaction with Phe1 (b in Fig. 8E). In DFT calculations (see Fig. S5, ESI†), the Phe2–Phe1 CH–π interaction (b) is 12.9 kcal mol^−1^ more stable than the conformation with only a close Phe2–Ni^2+^ contact (a). The conformation with a Phe1–Ni^2+^ cation–π interaction is 9.2 kcal mol^−1^ more stable than the weaker close Phe2–Ni^2+^ contact [note: previous DFT calculations^89^ that identified a cation–π interaction for only Phe2 erroneously used the wrong chirality for Phe1 which prevented the formation of the Phe1⋯Ni^2+^ interaction observed here]. In simulations of P3, Phe1 is prevented from forming close contacts with the nickel center due to steric interactions with Ile2. The formation of cation–π interactions with Ni^2+^ could contribute to the lack of change in the insertion depth of the N-terminal end (*z*_N_ ∼ 9.1 Å) when Phe2 (in P1:Ni) is replaced by Ile2 (in P3:Ni). Similar cation–π interactions, if present in cuprated piscidins, could also contribute to the lower nuclease activity^26^ of the P1:Cu^2+^ relative to P3:Cu^2+^ if the interactions protect the metal from redox events.

## Conclusions

The novel antimicrobial and antiviral therapeutic properties of piscidins are enhanced by metal binding to its ATCUN motif. All-atom molecular dynamics simulations examined the effect of metalation and oxidized lipid incorporation on the structural properties of the embedded peptides. Piscidins are found at distinct depths dependent on the isoform (P1 *vs.* P3) and state (free or Ni^2+^-bound). *Apo* P1 tends to insert deeper than P3 with no preference for a particular terminal end, while the N-termini of Ni^2+^-bound piscidins become more tilted into the bilayers due to charge neutralization of the ATCUN motif, confirming earlier predictions. Metal coordination also constrains the conformation dynamics of the N-terminus to position the nonpolar ATCUN sidechains in the lipid sublayer and His3 toward the headgroup sublayer. Cation–π interactions between Phe1 and the bound Ni^2+^ and CH–π interactions between Phe1 and Phe2 are found intermittently in the P1:Ni simulation and may contribute to protection against redox events at the metal center. These interactions in metalated P3 are blocked by the bulky sidechain of Ile2, suggesting a potential mechanism for the observed differential antimicrobial properties of metalated P1 and P3. This sequence dependence could be harnessed for engineering of new antimicrobial piscidins. Piscidins induce expansion and increased fluidity of the bilayer that could precede complete membrane disruption and bacteria death, but larger leaflet models are required to observe these events. Chain reversal of the oxPL chains into the aqueous phase reduces the number of hydrogen bonds between the headgroup constituents and may contribute to disruption of the bulk properties of the aldo-PC-containing membrane.

## Conflicts of interest

There are no conflicts to declare.

## Supplementary Material

CB-004-D3CB00035D-s001
