# Supplementary material for: The effect of metalation on antimicrobial piscidins imbedded in normal and oxidized lipid bilayers
Source: RSC Chem Biol. 2023 Jun 7;4(8):573–86. doi: 10.1039/d3cb00035d (PMC10398361; doi:10.1039/d3cb00035d)
Supplement: CB-004-D3CB00035D-s001 [file CB-004-D3CB00035D-s001.pdf]

Electronic Supplementary Material (ESI) for RSC Chemical Biology.
This journal is © The Royal Society of Chemistry 2023

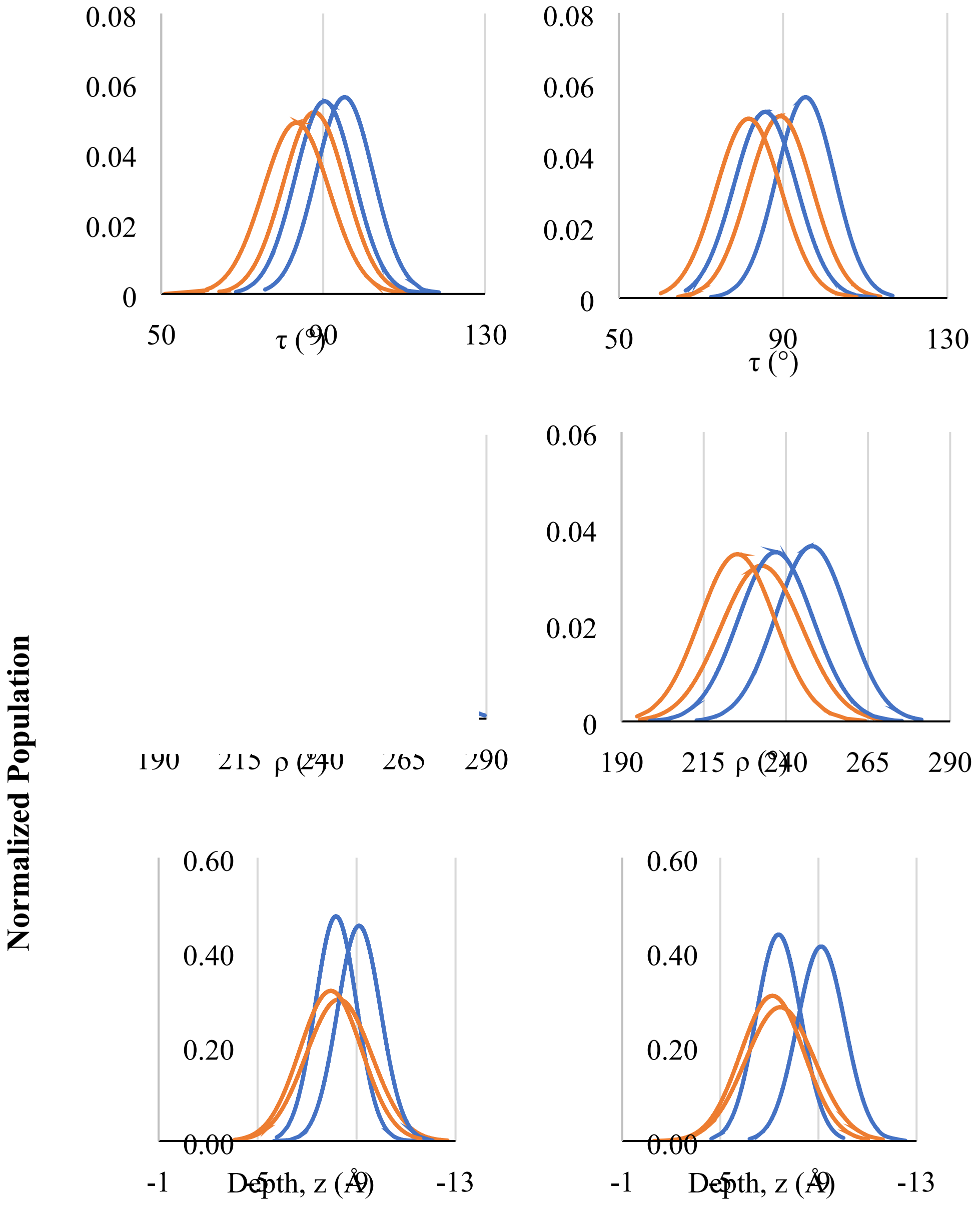

Figure S1. Distribution of tilt angles (τ), azimuthal rotation angles (ρ), and depths of insertion sampled by the N- (blue) or C- (orange) terminal residues of P1 (left) or P3 (right) in POPC/POPG during the 2-µs MD simulations. Curves for *apo* and metalated piscidins are shown as dashed and solid lines, respectively.

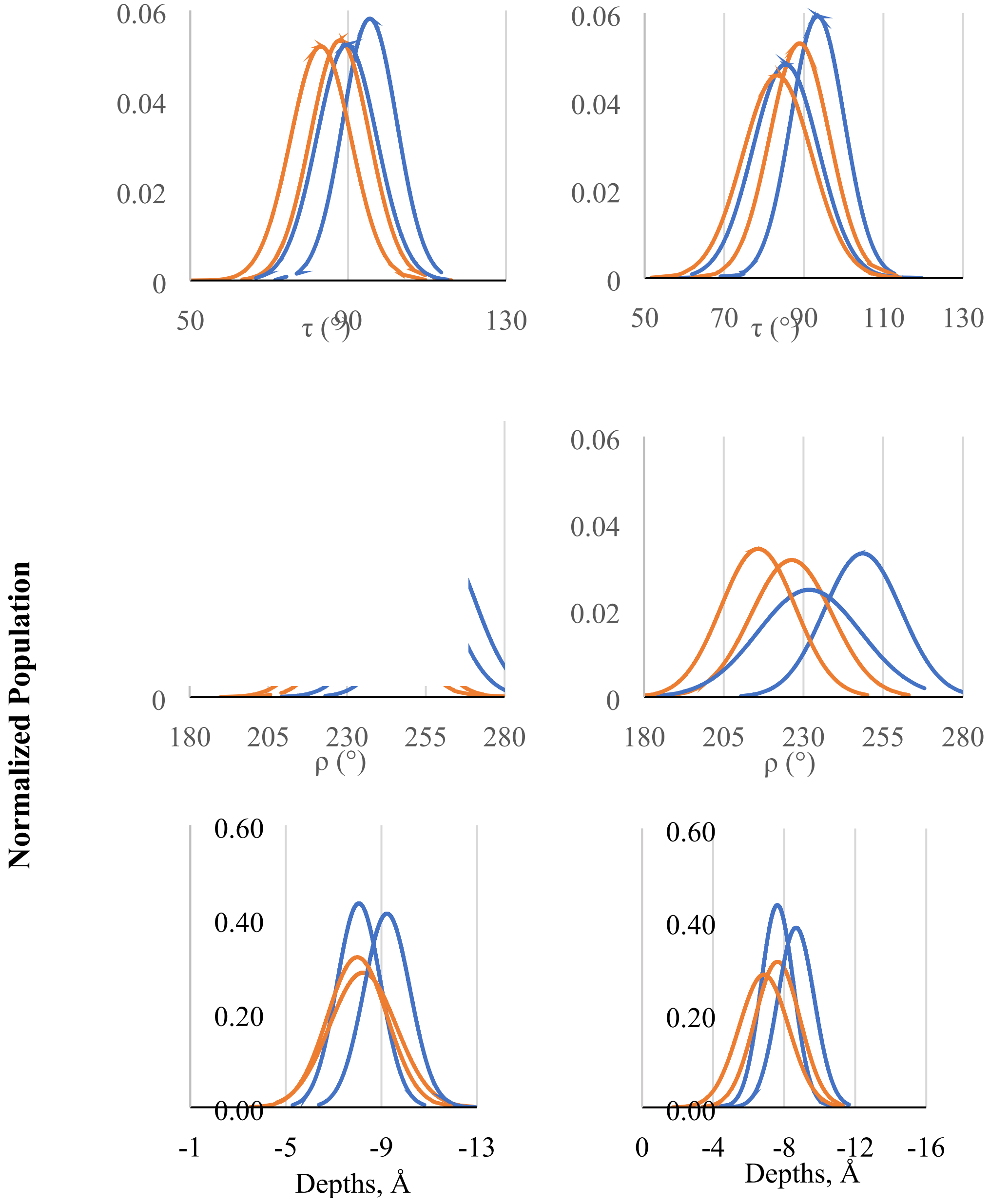

Figure S2. Distribution of tilt angles, azimuthal rotation angles, and depths of insertion sampled by the N- (blue) or C- (orange) terminal residues of P1 (left) or P3 (right) in POPC/POPG/aldo-PC during the 2-µs MD simulations. Curves for *apo* and metalated piscidins are shown as dashed and solid lines, respectively.

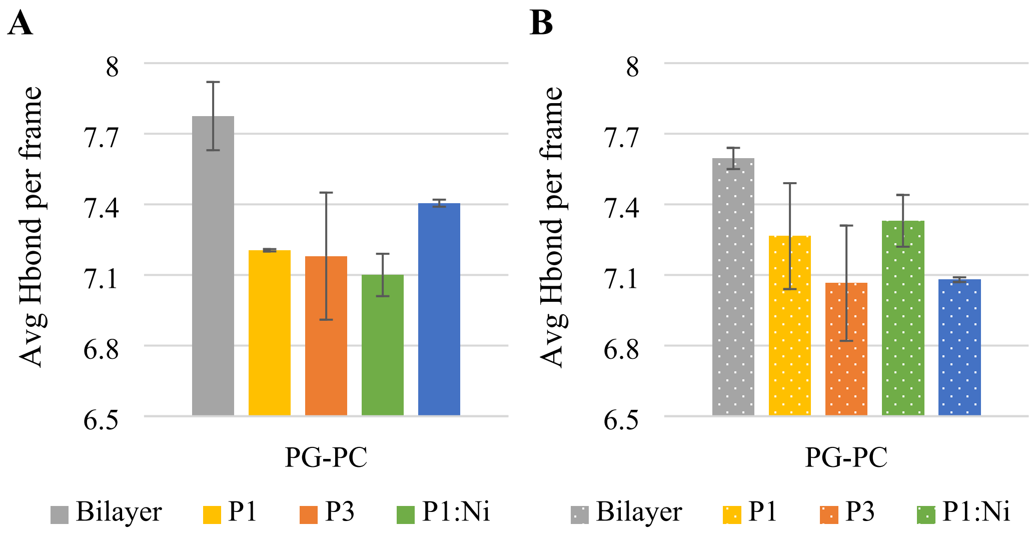

Figure S3. Average hydrogen bonds per frame between PC and PG headgroups in (A) POPC/POPG and (B) POPC/POPG/aldo-PC bilayer.

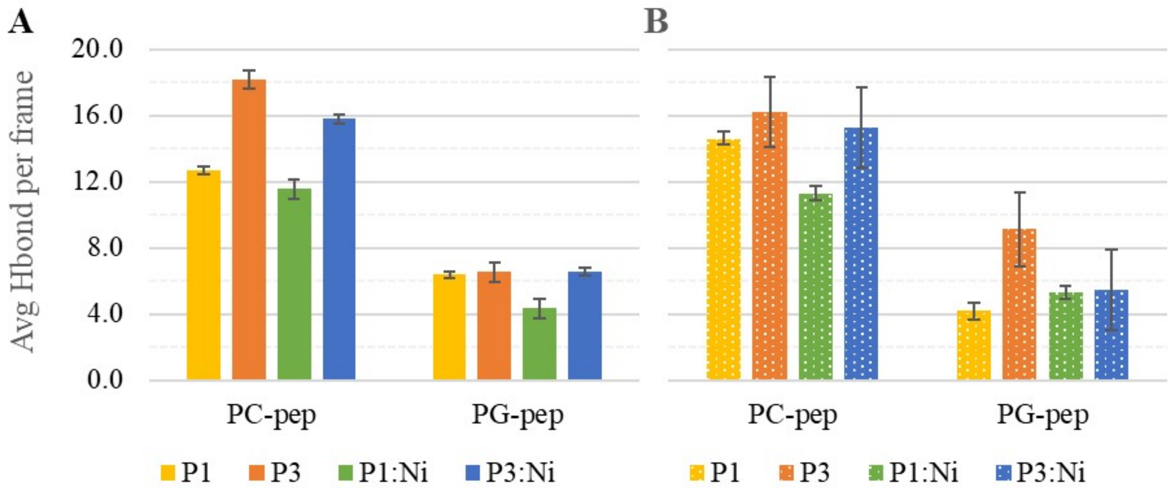

Figure S4. Average hydrogen bonds per frame between piscidin and lipid headgroup constituents in (A) POPC/POPG and (B) POPC/POPG/aldo-PC bilayer.

Density functional theory calculations

Density functional theory (DFT) calculations were performed on the P1 ATCUN motif using Gaussian09[1] and the M06-2X[2] exchange correlation functional. Snapshots extracted from MD trajectories were stripped of solvent, truncated to Ile5, and capped with a methylamine group. Nickel was represented with

the Wachters-Hay all-electron basis set with all other atoms using the TZVP basis set.[3,4] Geometry optimizations were performed using the integral equation formalism polarizable continuum model (IEF-PCM) with heptane as the solvent.[5]

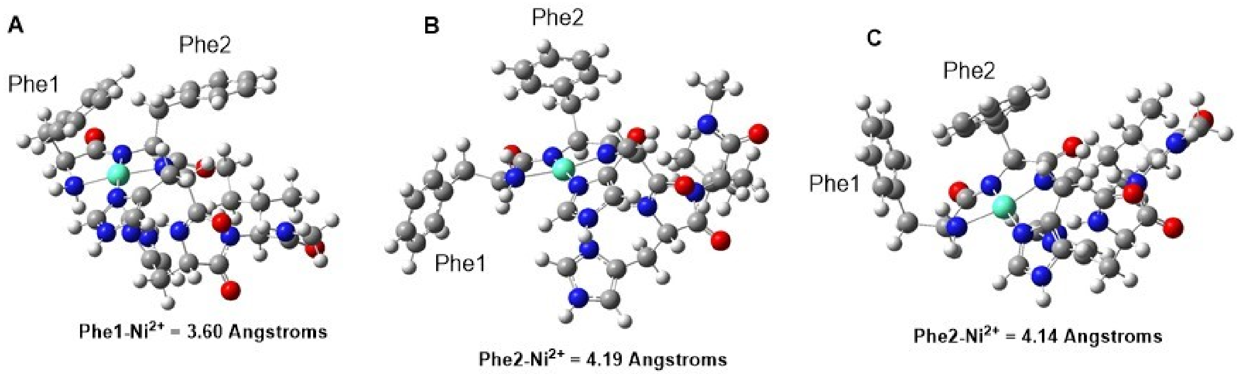

Figure S5. DFT-optimized structures of representative snapshots of Phe1- and Phe2-Ni^{2+} close contacts. Relative energies are calculated with respect to C. (A) Phe1 forms a short interaction consistent with a cation-π interaction between Phe1 and the metal ion (+3.7 kcal/mol). The longer close contact between Phe2 and the metal ion in B is higher in energy (+12.9 kcal/mol) than the conformation in which the Phe2-Ni^{2+} close contact is supported by a CH-π interaction between Phe2 and Phe1 (0.0 kcal/mol).

References

1 Gaussian 09 Gaussian, Inc., Wallingford, CT 2009.
2 Y. Zhao and D. G. Truhlar, *Theor. Chem. Acc.*, 2008, **120**, 215–241.
3 A. J. H. Wachters, *J. Chem. Phys.*, 1970, **52**, 1033–1036.
4 P. J. Hay, *J. Chem. Phys.*, 1977, **66**, 4377–4384.
5 J. Tomasi, B. Mennucci and R. Cammi, *Chem. Rev.*, 2005, **105**, 2999–3094.
